# Removal of an endobronchial lipoma via uniportal thoracoscopic right basal segmentectomy

**DOI:** 10.1186/s40792-020-00973-z

**Published:** 2020-08-17

**Authors:** Tomohiro Yazawa, Hitoshi Igai, Natsumi Matsuura, Fumi Ohsawa, Mitsuhiro Kamiyoshihara

**Affiliations:** Department of General Thoracic Surgery, Japanese Red Cross Maebashi Hospital, 389-1, Asakura, Maebashi, Gunma 371-0811 Japan

**Keywords:** Endobronchial lipoma, Surgery, Segmentectomy, Lipoma, Bronchial obstruction

## Abstract

**Background:**

Endobronchial lipoma is a rare benign tumor. The standard treatment is bronchoscopic intervention or surgical resection. Here, we present a case of endobronchial lipoma treated by right basal segmentectomy via a uniportal thoracoscopic approach.

**Case presentation:**

A 66-year-old female presented with a persistent cough and recurring high-grade fever 4 months in duration. Chest X-ray revealed an abnormal infiltration shadow in the right lower lung field. Chest computed tomography revealed a tumor that occluded the lateral segmental bronchus of the right lower lobe. Surgical resection was planned because we failed to diagnose the tumor via bronchoscopic biopsy. Finally, uniportal, thoracoscopic right basal segmentectomy, which is less invasive than lobectomy and allows preservation of the apical segment of the lower lobe, was successfully performed. The pathological diagnosis was of endobronchial lipoma. The cough and recurrent pneumonia improved after surgery.

**Conclusions:**

Segmentectomy via a uniportal thoracoscopic approach could be a novel treatment option for endobronchial lipoma.

## Background

Endobronchial lipoma, first reported by Carl von Rokitansky in 1854, is extremely rare, constituting only 0.1–0.5% of all lung tumors and 4.6% of all benign lung tumors [1, 2]. Occasionally, endobronchial lipoma triggers repeated pneumonia because it causes bronchial obstruction (depending on the tumor size). In such cases, the tumor must be removed via a bronchoscopic or surgical approach to prevent damage to the lung parenchyma. Bronchoscopic resection is the first-choice treatment, because it is less invasive than surgical resection. However, surgical resection may be the only option if it is technically difficult to diagnose or remove the tumor bronchoscopically. When planning resection of such a benign tumor, the less invasive uniportal thoracoscopic approach is preferable. Here, we present a case of endobronchial lipoma treated by right basal segmentectomy via a uniportal thoracoscopic approach.

## Case presentation

**Fig. 1:**
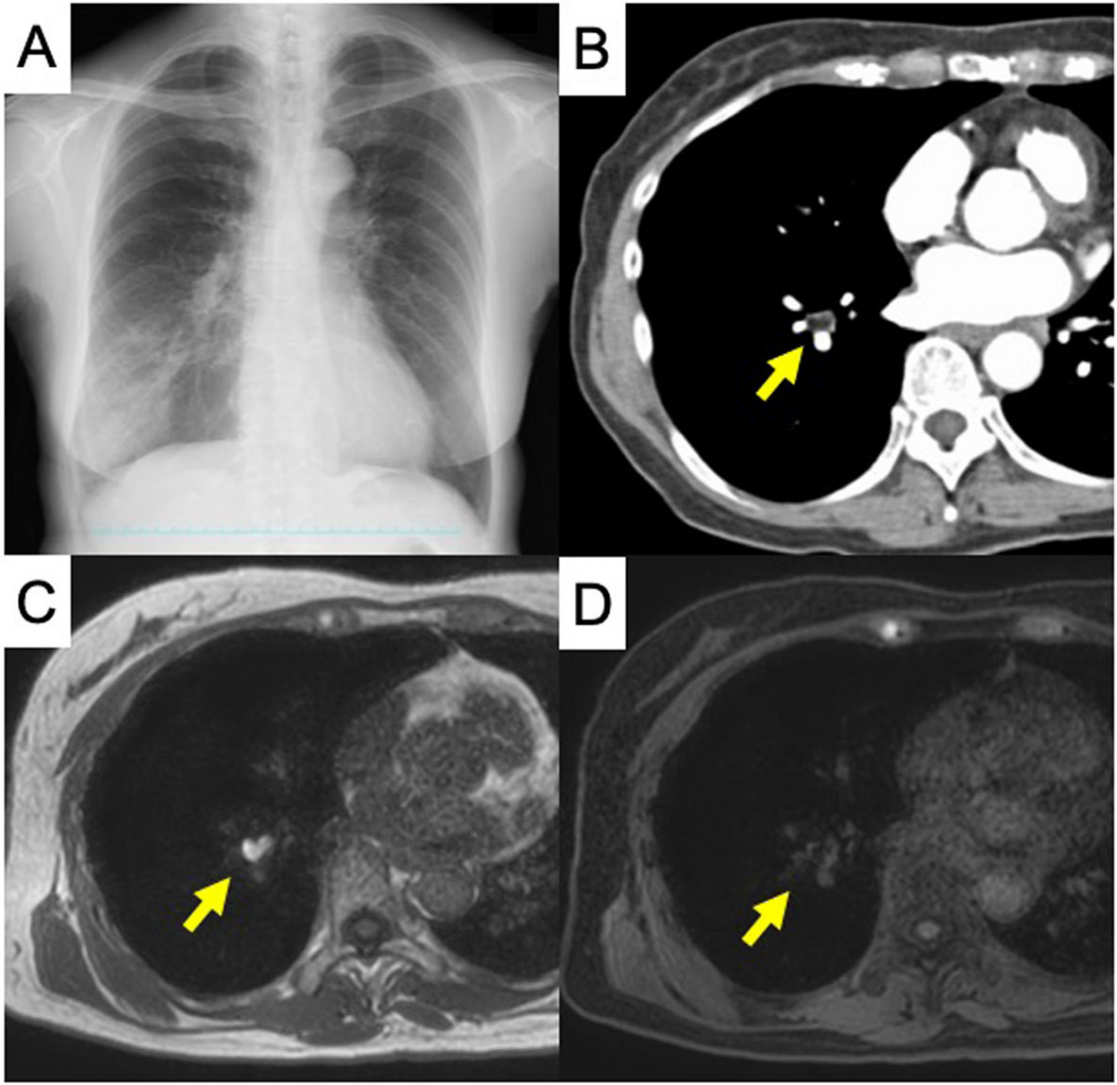
Preoperative imaging test. **a** Chest X-ray showing a shadow on the right lower lung field. **b** Computed tomography image showing a low-density nodule without calcification occluding the lumen of the lateral segmental bronchus of the right lower lobe (B^9^). **c** Magnetic resonance image showing a nodule of high intensity on T1-weighted images and **d** low intensity on fat suppression images

**Fig. 2:**
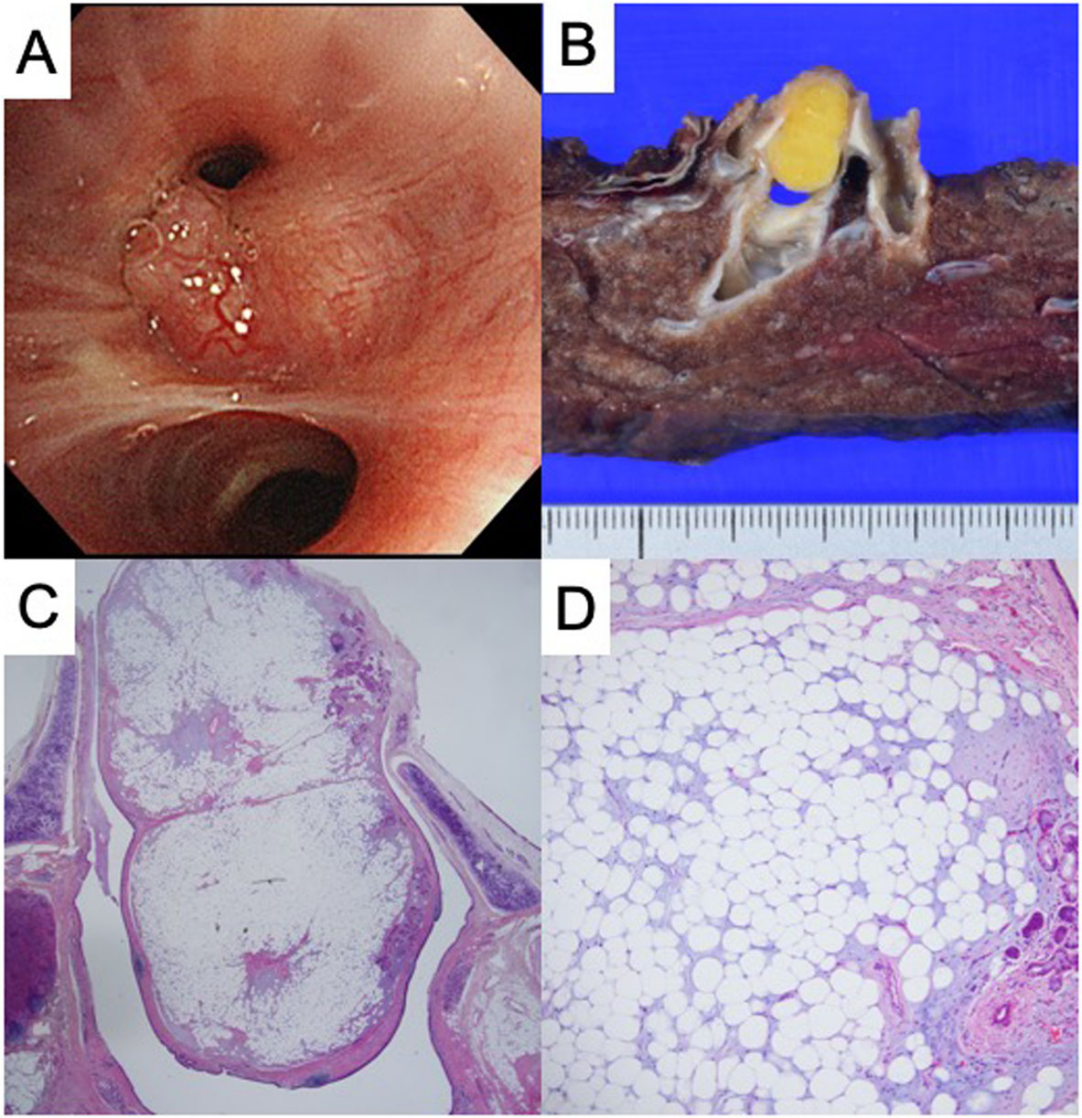
Pathological findings. **a** Bronchoscopy findings of a relatively well-defined, smooth tumor that fully occluded the lumen of the lateral segmental bronchus of the right lower lobe (B^9^). **b** Macroscopic examination of the right basal segmental specimen showing that the bronchial tumor originated from the entrance of the lateral segmental bronchus of the right lower lobe (B^9^). **c** Microscopically, the tumor lay inside the bronchial cartilage and **d** consisted principally of mature adipose tissue


A 66-year-old female with a persistent cough and recurring high-grade fever 4 months in duration presented to our hospital. The patient had no smoking or medical history. A chest radiograph revealed an infiltrative shadow in the right lower lung field (Fig. 1a), and chest computed tomography (CT) revealed a low-density 11-mm nodular shadow without calcification that fully occluded the internal lumen of the lateral segmental bronchus of the right lower lobe (B^9^) (Fig. 1b). Magnetic resonance imaging (MRI) revealed a nodule exhibiting high intensity on T1-weighted images and low intensity on fat suppression images (Fig. 1c, d). Bronchoscopy revealed an occluded lateral segmental bronchus with an endobronchial tumor (Fig. 2a). However, we failed to obtain a specimen volume adequate for diagnosis because the tumor bled readily. Therefore, we planned surgical resection to confirm the diagnosis and prevent pneumonia recurrence. We scheduled uniportal thoracoscopic right basal segmentectomy, which is less invasive than lobectomy and preserves the apical segment of the lower lobe (B^6^). The operation was performed using one-lung ventilation with the patient under general anesthesia in the lateral decubitus position. A 4-cm access incision was made in the fifth intercostal space of the anterior axillary line. Intraoperative findings revealed a dense hilum due to inflammatory changes caused by the repeated pneumonia. The duration of surgery was 170 min, and the blood loss was 50 ml (supplementary video). Histology confirmed endobronchial lipoma, and that the resected specimen had negative margins (Fig. 2b–d). The chest drainage tube was removed on postoperative day 1. The patient discharged on postoperative day 2 and remains alive, without cough or pneumonia.

**Additional file 1. Supplementary video.** The uniportal thoracoscopic right basal segmentectomy. Intraoperative findings revealed a dense hilum due to inflammatory changes caused by the repeated pneumonia.

## Discussion

Endobronchial lipoma is more frequent in males than females; the mean patient age is 60 years. The tumor shows a preference for an airway lobar or subsegmental location and is more frequent on the right side, as in our case [1]. Common clinical symptoms include cough, wheezing, intermittent shortness of breath, sputum production, and fever caused by bronchial obstruction. Such symptoms may result in misdiagnosis of asthma or chronic obstructive pulmonary disease. CT and MRI are useful for diagnostic confirmation. Typically, CT reveals a homogenous area of fat density that is not contrast-enhanced, while MRI may reveal a mass of high intensity on T1-weighted images but low intensity on fat suppression images [3].

We failed to diagnose the tumor bronchoscopically because insufficient material was obtained; the tumor bled readily. Elsayed et al. failed to diagnose endobronchial lipoma via flexible bronchoscopy for the same reason and reported that large excisional biopsy was optimal for diagnosing benign tumors such as endobronchial lipomas [4]. Nussbaumer et al. reported that endobronchial lipomas mimicked bronchial carcinoid tumors [5]; thus, there is a need for differential diagnosis because the latter tumors may exhibit low-grade malignancy. Surgical resection was considered desirable if diagnosis via bronchoscopic biopsy failed. We considered segmentectomy preferable to bronchoscopic resection in our case.

Muraoka et al. recommended surgical resection of endobronchial lipomas under the following conditions: (1) difficulty in obtaining a definitive diagnosis along with a possibly malignant tumor, (2) peripheral destructive lung disease caused by long-term atelectasis or pneumonia, (3) presence of an extrabronchial growth or subpleural lipomatous disease, and (4) expected technical difficulties during bronchoscopy due to the presence of multiple tumors [1]. Unfortunately, we failed to diagnose the tumor bronchoscopically due to bleeding of the tumor. Therefore, we could not deny the possibility of carcinoid tumor. Additionally, the patient suffered from repeated pneumonia although her lung was not destructive. We considered these findings almost met with the 1st and 2nd criteria Muraoka proposed. In their review, no patients with endobronchial lipoma underwent pulmonary segmentectomy; lobectomy was the preferred surgical approach. However, the review did not mention which the surgical approach is selected thoracotomy or thoracoscopy. To minimize invasiveness, preserve the lung, and reduce postoperative pain, segmentectomy or a uniportal approach is desirable when we choose surgical resection for this benign disease. To the best of our knowledge, only Galvez et al. has reported successful use of uniportal pulmonary segmentectomy to treat a patient with an endobronchial lipoma [6]. Here, we successfully performed right basal segmentectomy via a uniportal thoracoscopic approach despite encountering a severe pleural adhesion and dense hilum. This minimally invasive surgical procedure contributed to the rapid recovery of our patient.

## Conclusion

In conclusion, segmentectomy via a uniportal thoracoscopic approach is a useful novel surgical option for endobronchial lipoma, associated with rapid recovery and preservation of respiratory function.

## Data Availability

Not applicable.

## Abbreviations

CT: Computed tomography
MRI: Magnetic resonance imaging
